# The Transitions Between Dynamic Micro-States Reveal Age-Related Functional Network Reorganization

**DOI:** 10.3389/fphys.2018.01852

**Published:** 2019-01-04

**Authors:** Yuanyuan Chen, Ya-nan Liu, Peng Zhou, Xiong Zhang, Qiong Wu, Xin Zhao, Dong Ming

**Affiliations:** ^1^College of Microelectronics, Tianjin University, Tianjin, China; ^2^Tianjin International Joint Research Center for Neural Engineering, Academy of Medical Engineering and Translational Medicine, Tianjin University, Tianjin, China; ^3^Department of Biomedical Engineering, College of Precision Instruments and Optoelectronics Engineering, Tianjin University, Tianjin, China

**Keywords:** dynamic functional connectivity, normal aging, micro-state, resting-state fMRI, network reorganization

## Abstract

Normal dynamic change in human brain occurs with age increasing, yet much remains unknown regarding how brain develops, matures, and ages. Functional connectivity analysis of the resting-state brain is a powerful method for revealing the intrinsic features of functional networks, and micro-states, which are the intrinsic patterns of functional connectivity in dynamic network courses, and are suggested to be more informative of brain functional changes. The aim of this study is to explore the age-related changes in these micro-states of dynamic functional network. Three healthy groups were included: the young (ages 21–32 years), the adult (age 41–54 years), and the old (age 60–86 years). Sliding window correlation method was used to construct the dynamic connectivity networks, and then the micro-states were individually identified with clustering analysis. The distribution of age-related connectivity variations in several intrinsic networks for each micro-state was analyzed then. The micro-states showed substantial age-related changes in the transitions between states but not in the dwelling time. Also there was no age-related reorganization observed within any micro-state. But there were reorganizations observed in the transition between them. These results suggested that the identified micro-states represented certain underlying connectivity patterns in functional brain system, which are similar to the intrinsic cognitive networks or resources. In addition, the dynamic transitions between these states were probable mechanisms of reorganization or compensation in functional brain networks with age increasing.

## Introduction

Normalbrain aging refers to the degradative phenomena that occurs in brain structure, function and morphology with age increasing and manifests as a certain degree of brain dysfunction in elderly populations (Hedden and Gabrieli, 2004; Whalley et al., 2004). As the aging population issue is becoming increasingly serious, diseases characterized by cognitive dysfunction, such as Alzheimer’s disease (AD), Parkinson’s (PD) and other neurodegenerative diseases, are also increasing at an unprecedented rate (Reeve et al., 2014; Xu et al., 2015). Currently, the mechanism of aging and even how our brain changes during aging are not clear.

Traditionally, resting-state functional connectivity, derived from the blood oxygenation level dependent (BOLD) signal over an entire scan (5 min or longer), was considered to be constant throughout the entire scan time. However, research has shown temporal dynamics of functional connectivity in resting state (Chang and Glover, 2010; Hutchison et al., 2013a; Feng et al., 2015; Leonardi and Van De Ville, 2015). This kind of dynamic functional connectivity, which varies over a matter of seconds, may be highly related to unconstrained mental activities (Hutchison et al., 2013b; Allen et al., 2014; Zalesky et al., 2014), as well as to neurologic diseases (Kaiser et al., 2015; Mayer et al., 2015; Braun et al., 2016; Wee et al., 2016). The time-varying functional connectivity derived from sliding window correlation, which reflects the dynamics of functional brain networks, is also expected to facilitate our understanding of the mechanisms of aging process. Qin et al. (2015) used amplitude of low-frequency fluctuations (ALFF) in dynamic functional connectivity to predict individual brain maturity in adolescents between 7 and 30 years of age and found that the internetwork exhibited substantial dynamic interaction changes that were highly associated with brain maturation. Chen et al. (2017) used dynamic functional connectivity (FCV) method to obtain index of dynamic functional connectivity variability and found that the variation of the spontaneous fluctuations in functional connectivity was highly related to brain aging process. However, these indexes of dynamic functional connectivity were time-averaged features that could not capture the connectivity patterns of the spontaneous fluctuations.

Recent studies have shown that connectivity dynamics can be captured as recurring patterns of connectivity or interactions among intrinsic networks during both tasks and resting (Hutchison et al., 2013a,b; Allen et al., 2014; Calhoun et al., 2014). In some studies (Allen et al., 2014; Calhoun et al., 2014; Rashid et al., 2014; Gonzalez-Castillo et al., 2015), these series of inherent functional connectivity patterns were called micro-states. The existence of micro-states of neural activity, which appeared early in electrophysiological studies (Lehmann, 1990; Pascual-Marqui, 1995), was also considered a probable interpretation for the spontaneous fluctuations in functional connectivity. Allen et al. (2014) firstly described a data-driven approach to reveal these functional states and suggested that the time-varying aspects of functional states can unveil the functional coordination among different neural systems. Many recent studies supported this claim and reported positive evidence that the functional micro-states could provide valuable insights into the pathophysiological changes of the brain (Rashid et al., 2014; Gonzalez-Castillo et al., 2015; Du et al., 2016).

Prior studies revealed functional decline and compensation in human brain occurring with normal aging (Reuter-Lorenz and Campbell, 2008). In fact, with age increasing, brain functional connectivity or networks also show dynamic changes (Park and McDonough, 2013). For example, in aging individuals, the primary perceptual system declines, and higher-order cognitive systems are recruited to offset deficits in sensory processing (Davis et al., 2008; Vinette and Bray, 2015); the decay in the default mode network can lead to decreased anticorrelations and increased regulation from the frontoparietal control system (Grady et al., 2016; Spreng et al., 2016). These results indicates that frequent changes in internetwork interactions and some connectivity transition patterns are associated with aging. We expected that the dynamic changes between connectivity states could offer important insights related to understanding the functional reorganizations that occurs with aging. Considering that the functional connectivity patterns reoccur across windows and subjects, they would likely provide valuable insight regarding both the mechanism of functional connectivity fluctuations on the scale of seconds and the intrinsic reorganizations of functional networks that occurs during aging.

The aim of this study was to reveal that how brain dynamic functional networks change with age increasing. We first applied K-means clustering to extract the common functional connectivity states and then extracted the connectivity states for each subject. We evaluated the differences in the dynamic connectivity patterns between groups, including the young group, the adult group and the old group. Our major goals involved the following aspects: (1) whether the temporal transitions among functional micro-states alter with age increasing; (2) whether there exists certain specific intrinsic connectivity pattern that functional states exhibit during the aging process; and (3) whether the three different age groups would demonstrate differing strengths of the functional connectivity states.

## Materials and Methods

### Participants

Experimental data on 86 healthy subjects were obtained from an open database, namely, the Nathan Kline Institute Rockland Sample (NKI-RS), published by the Nathan Kline Institute^1^. All approvals and procedures for collection and sharing of data were approved by the NKI institutional review board, and each participant was gave written informed consent. We divided the subjects into three groups: the young group (Y: *n* = 31, mean age = 26.2 years, range = 21–32 years), the adult group (A: *n* = 28, mean age = 46.1 years, range = 41–54 years), and the old group (O: *n* = 27, mean age = 68.4 years, range = 60–86 years). There were significant differences in age but not in gender or hand dominance.

### Data Acquisition

All the data were obtained using a Siemens 3.0 T Trio Tim magnetic resonance scanner, approved by the relevant institutional review board, using an echo-planar imaging (EPI) sequence. During scanning, all subjects were asked to maintain a resting state, that is, to stay relaxed with their eyes closed, to move as little as possible, and to not engage in any brain activities. The scanning parameter settings of the resting-state fMRI were as follows: repetition time (TR) = 2500 ms, echo time (TE) = 30 ms, flip angle (FA) = 80, field of view (FOV) = 216 mm × 216 mm, voxel size = 3 mm × 3 mm × 3 mm, slices = 38, scan time = 650 s, and time points = 260. All participants also underwent a 3D high-resolution T1 sequence: TR = 2500 ms, TE = 3.5 ms, FA = 8°, FOV = 256 mm× 256 mm, resolution = 1 mm× 1 mm× 1 mm, and 192 slices.

### Data Pre-processing

The data need to be pre-processed to eliminate the noise caused by head movement, respiration and other factors with DPABI v3.1 (Yan et al., 2016; a toolbox for Data Processing and Analysis for Brain Imaging^2^) with is fMRI processing platform based on SPM (Statistical Parametric Mapping^3^) embedded in MATLAB. First, to ensure that the magnetization reached a steady state and that the subjects adapted to the scanning environment, the first four timepoints of each fMRI scan were discarded. The remaining 256 timepoints were corrected for the time layer. After this correction, all layers were estimated as being obtained at the same time. Taking into account that subjects will unconsciously move their heads, the head motion of the subjects was estimated with Friston 24-parameter correction (Yan et al., 2013). Then, nuisance terms were removed from the resting-state BOLD time series through multiple linear regression. These nuisance regressors included: linear and quadratic trends, 24 motion parameters estimated during image co-registration, and the mean BOLD signal calculated from WM and CSF regions, where these regions were defined using partial volume thresholds of 0.99 for each tissue type and erosion of two voxels in each direction to minimize partial voluming with gray matter. Then, the fMRI data were warped to the Montreal Neurological Institute (MNI-152) template and resampled to 3 mm × 3 mm × 3 mm isotropic voxels. In this paper, we used a 0.01–0.08 Hz bandpass filter to obtain extremely low-frequency neural signals, as it is generally believed that the functional magnetic resonance signals in this frequency band best reflect neural activity. Finally, spatial smoothing was performed with a Gaussian filter kernel (FWHM = 6 mm) to reduce registration errors and individual differences between subjects. All the subjects were checked for head motion; the mean displacement was <2.5 mm and the mean rotation was <2.5°. Any subject with bigger displacement or rotation, If only for one volume ever the scan time, has been removed. There was no significant difference in mean head motion between groups based on the subject-averaged framewise displacement (FD) measurement (Power et al., 2011; Yan et al., 2013) : *Y* = 0.090 ± 0.037; *A* = 0.091 ± 0.028; *O* = 0.10 ± 0.036; ANOVA test: *F*(2, 85) = 1.890; *P* = 0.339.

### Dynamic Functional Connectivity

Networks were defined in the Montreal Neurological Institute (MNI) space according to Dosenbach et al. (2011), including 160 regions and six intrinsic sub-networks: the CON (cingulo-opercular network), DMN (default mode network), FPN (frontoparietal network), OCC (occipital network), SMN (sensorimotor network), and CER (cerebellar network). The BOLD signals of all voxels in each region, defined using a spherical region of interest (ROI) with a diameter of 10 mm, were averaged and extracted. A functional connectivity (FC) matrix was then estimated with the Fisher Z-transformed Pearson correlations of the BOLD signals between paired regions.

Sliding window correlation method (Sakoglu et al., 2010; Di and Biswal, 2013; Hutchison et al., 2013a) is a widely applied technique to observe the functional dynamics. Using this method, a functional connectivity matrix is calculated for every short window, which slides along the scanning time. Then, in this study, the dynamic FC was estimated using the sliding window correlation method, and 160 × 160 × N FC matrix sequences were obtained for each subject. We used a fixed-length rectangular window (width = 24 × TR = 60 s; thus, *N* = 232), and the window was shifted by one TR. The Fisher Z-transformed Pearson correlations were also used here for the FC. The calculating of dynamic functional connectivity is finished with in-house programming in MATLAB (R2016a) according to the methods stated in previous papers (Sakoglu et al., 2010; Di and Biswal, 2013; Hutchison et al., 2013a).

### Clustering Analysis

**FIGURE 1:**
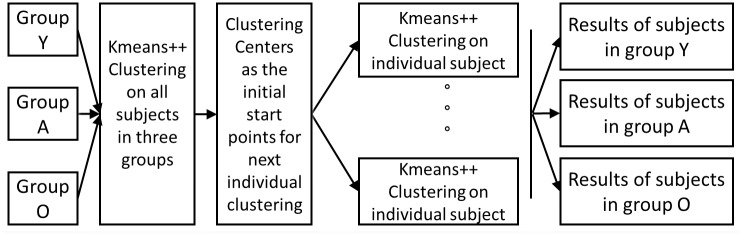
The working flow of the clustering analysis. Firstly perform k-means++ clustering on all subjects in three groups, and the resulted clustering centers as the numbers were used as the initial starts for the individual k-means++ clustering; for all the results of individual clustering results, we observed the group-level difference.

For each subject, the windowed functional connectivity matrixes with high variability were selected as subject exemplars, and used in K-means clustering to obtain global clustering centers. The K-means plus (k-means ++) algorithm was utilized here to perform clustering analysis. We repeated the clustering method using different distance functions (correlation and cosine rather than the L1-norm) and found similar results; thus, we used the correlation distance in this paper. We determined the optimized number of clusters to be five using the elbow criterion of the cluster validity index, which was computed as the ratio of the within-cluster distances to the between-cluster distances. These global clustering centers were then used as starting points to cluster the dynamic functional connectivity matrix data for each subject. Subsequently, the median of all the matrixes classified into each center was calculated as the representation of one micro-state. This workflow of the clustering analysis is illustrated in Figure 1.

There were two basic indexes used to evaluate the transitions between micro-states. First, the dwelling time, which was represented by the times or occurrence time of one state throughout the entire scanning time, was calculated as the number of matrixes classified into this state and represented the occurrence probability of each state. Second, the transition time, which represented the probability of transition from the current state to another, was calculated as the number of transitions from one state to another. Here, the time could be represented as a percentage of the total scanning time.

### Statistical Analysis

Subsequently, based on the subject-specific states, we compared the three groups with respect to the dwelling time of each state, the transition time between states and the functional connectivity strength in the corresponding states. After a normality test and a homogeneity test of variance, a two-sample *t*-test was utilized on the dwelling time and the functional connectivity in each state, and nonparametric permutation test was utilized on the transition time between states with 1,000 times of randomization. To distinctly assess the age-related changes of the functional connectivity strength in each micro-states, the network averaged functional connectivity was calculated using the significant connections, which were identified by one sample *t*-tests within each group and each state with an FDR-corrected (false discovery rate) *p* < 0.05. Then, one-way ANOVA tests were to reveal the significant age related changes of network connectivity (FDR-corrected *p* < 0.05). The F-statistics were used and averaged to access the age related variability for each sub-network in each state.

## Results

### The Dwelling Time of the Functional Micro-States

**FIGURE 2:**
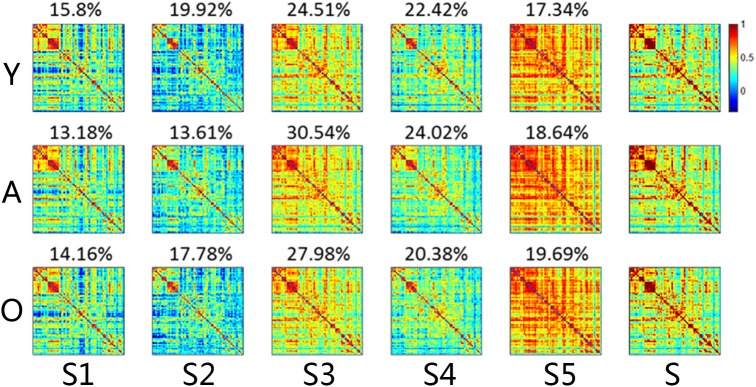
The connectivity matrix and averaged dwelling time of the micro-states for each group, including both dynamic and stationary matrixes. The color represents the level of functional connectivity according to the color bar. Y, young group; A, adult group; O, old group. The percentages on the matrixes are the transition time percentages of the state in each group. Color bar here represented the *T*-value.

**FIGURE 3:**
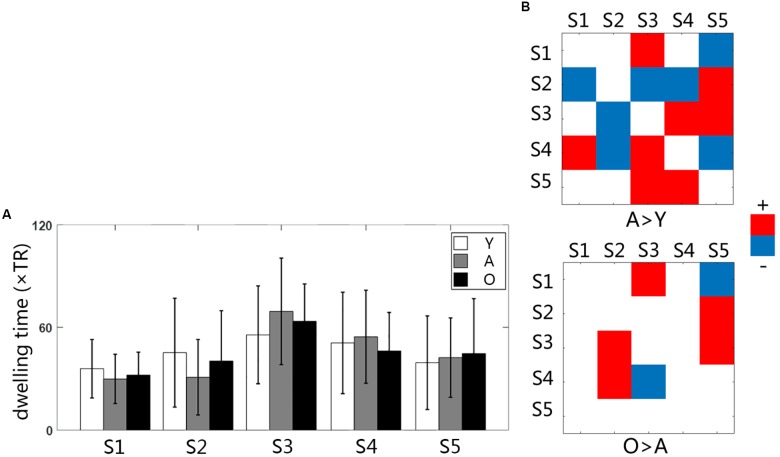
**(A)** The bar graph of averaged dwelling time for three groups; **(B)** the group differences of transition time between states Y, young group; A, adult group; O, old group.

We used the K-means clustering method to identify re-occurring patterns of FC states in each subject, and the group-averaged states and the group-averaged dwelling time are illustrated in Figure 2, including state 1 (S1), state 2 (S2), state 3 (S3), state 4 (S4), and state 5 (S5). Different states showed different connectivity patterns and strengths. For same states, the connectivity patterns were similar among the groups, but the dwelling times were not. On average, the dwelling time of state 3 was the highest among all three groups. However, there were no significant differences in the dwelling times of all states between the three groups (Figure 3A). The significant level here was FDR corrected *p* < 0.05.

### The Transition Time of the Functional Micro-States

**FIGURE 4:**
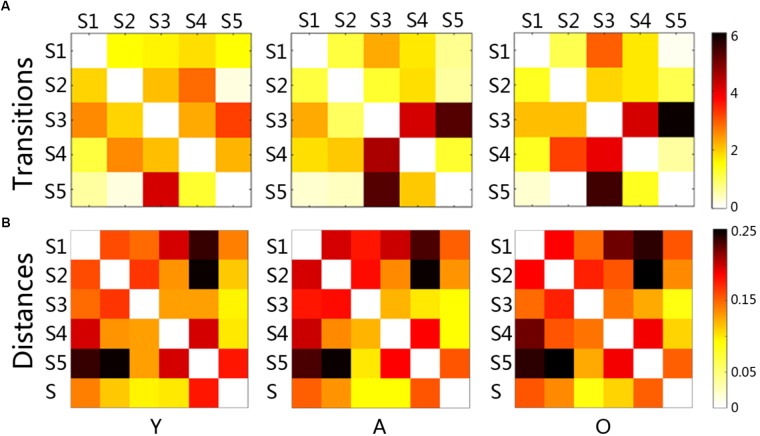
The matrixes of transition time and the distance matrixes between micro-states for each group **(A)**: each node (a little block) represented the averaged transition time between paired states, and the red and black colors indicate high transitions; **(B)**: each node (a little block) represented the distance between paired states, and the red and black colors meant large distance; Y, young group; A, adult group; O, old group.

Statistical comparisons indicated that the transition between states changed significantly with age increasing (Figure 3B). The changes between the young and the adult group showed some decreased transitions and other increased transitions (*p* < 0.05). The changes between the adult and the old group were less than that in the early development stage, indicating more increased transition than the decreased transition. The transition time between states was calculated in each subject, and the averaged transition matrixes for each group were represented in Figure 4A. The transition matrixes were almost symmetric, meaning that the transition was undirected. These transition matrixes showed clear patterns that were similar among the three groups. Distinct transition patterns and significant changes between groups could be observed in the transition matrixes. High-level transition frequencies were observed between S1 and S3, S2 and S4, S3 and S4, and S3 and S5. These transition patterns were stronger in the older groups than that in the young group. Additionally, Figure 4B shower the distances between all micro-states and the stationary connectivity. S3 and S4 showed the highest similarity (the lowest distance) with the stationary connectivity. Among the transitions between micro-states, S1–S3, S4–S2, S4–S3, and S5–S3 showed higher level of similarity (shorter distances) than the other transitions.

### Differences of Functional Connectivity in Each Micro-State

**FIGURE 5:**
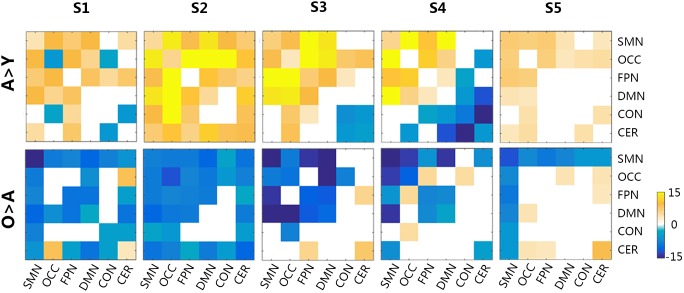
The group differences in functional connectivity across all micro-states. The level of significance was an FDR-corrected *q*-value < 0.05. Y, young group; A, adult group; O, old group; CON, cingulo-opercular network; DMN, default mode network; FPN, frontoparietal network; OCC, occipital network; SMN, sensorimotor network; CER, cerebellar network. Color bar represented the *T*-values.

**FIGURE 6:**
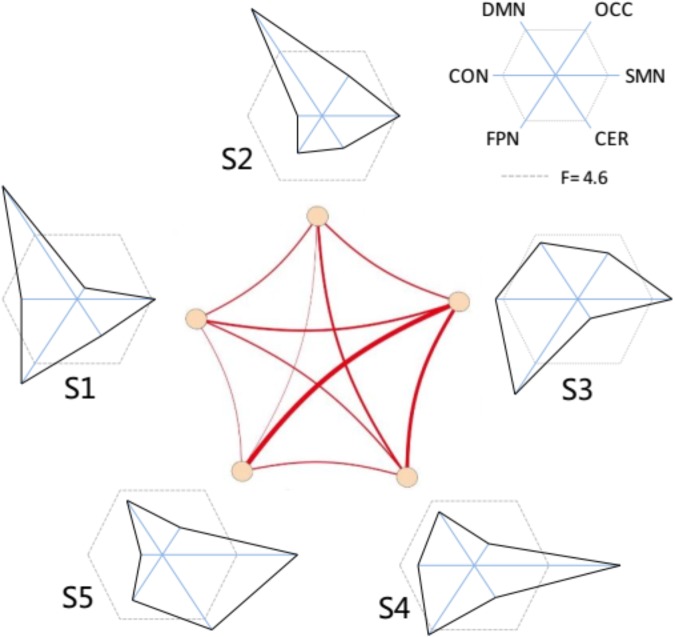
The variability distribution of the sub-networks for each micro-state. The center represent the averaged transitions between states. The radius of each solid line represents the *F*-values from the one-way ANOVA; the dotted lines represented line that *F*-value = 4.6 (inside is FDR corrected *p* > 0.001; outside is FDR corrected *p* ≤ 0.001).

The functional connectivity of all states showed substantial and significant changes between groups (Figure 5), with statistical level of FDR corrected *p* < 0.05. Almost all network connections showed inverted U shapes from the young to the old subjects, which meant increasing FC from the young subjects to the adult subjects and decreasing FC from the adult subjects to the old subjects. For example, the overall connections in S1 and S2 changed in this pattern. The FC of SMN, OCC, FPN, and DMN in S3 also showed inverted-U-shaped changes, and the connectivity of CON and CER decreased in the adult subjects. This same pattern was also observed in S4, as the CON-CER FC decreased in the adult subjects. However, the CON-associated FC and CER-associated internetwork FC also decreased in the adult group. Regarding S5, there were less significant changes, but the SMN and its associated networks showed similar inverted U-shaped changes. Figure 6 illustrated the level of age-related variability for all networks in each state based on the F statistics from ANOVA tests. The dotted line represented *F*(2, 85) = 4.60 (FDR corrected *p* < 0.001), based on which the solid line outside this dotted line showed dominant age related variability. Each state showed different age related variability for every sub-network. S1 and S2 showed higher variability in DMN and then FPN and lower variability in CON, CER, and OCC. S3, S4, and S5 exhibited distinct high variability in SMN. S3 also showed high variability in FPN, S4 showed high variability in FPN and S5 also showed high variability in CER. The three groups averaged transition matrix between these states was visualized in the center of Figure 6, which will be further addressed in the discussion section.

## Discussion

In this work, we extracted micro-states of dynamic functional connectivity networks with sliding window correlation and clustering analysis. To the best of our knowledge, it is the first to investigate aging associated human brain changes from the aspect of dynamic functional micro-states using resting-state fMRI. We compared the micro-states between three age groups and explored the specific distribution of age-related functional variations in all intrinsic sub-networks for each micro-state. The results of the current study converged into two main findings: (1) the transition time of the micro-states showed significant alterations between the age-related groups, but the dwelling time of each state did not significantly differ from each other; (2) the functional connectivity of the micro-states showed inverted-U-shaped changes across the three groups. The following discussion will focus on these findings and will make clear what these results indicate.

### Micro-States Indicated the Functional Resources

A previous study (Rashid et al., 2014) suggested that dynamic functional connectivity captured stable connectivity patterns which were not observed in stationary connectivity. These functional micro-states might be the inherent stable connectivity patterns in functional system of human brain. Although no significant difference in the occurrence probabilities of the micro-states was found between groups, the occurrence probability varied between states. This finding might suggested that the existence of micro-states was related to the inherent organization or dynamics of functional networks. The distances between each state and stationary connectivity matrix were relatively lower than the distance between these states. Relative to the other states, state 3 indicated the highest probability of occurrence and showed the shortest distance with the stationary connectivity matrix. This could imply that each micro-state might be intrinsically part of the stationary connectivity pattern, which was reflected by the low distance between micro-states and stationary state. The stationary connectivity is the most important stable connectivity pattern of the functional system, which can be regarded as a basic brain connection pattern of the dynamic functional fluctuations. The different occurrence probability of each state in the same group might be associated with the intrinsic meaning of each micro-state.

This kind of connectivity pattern was the intrinsic feature of each micro-state. Our results of the functional connectivity showed inverted-U-shaped changes for the most connections in the states with age increasing. For the functional connectivity comparisons, the adult group showed mostly higher connectivity than that of the young group, and the old group showed mostly lower connectivity than that of the adult group. These differences were not like the age-related changes in the stationary functional connectivity, which were inverted-U-shaped in intra-network connectivity and U-shaped in internetwork connectivity (Betzel et al., 2014; Cao et al., 2014; Keunen et al., 2017; Siman-Tov et al., 2017). It is well established that the intrinsic functional resources in our brain increase during maturation in the early years and decrease with the aging process. The functional connectivity within specific networks, such as the DMN (Betzel et al., 2014; Cao et al., 2014; Douaud et al., 2014; Geerligs et al., 2014), CON (Betzel et al., 2014; Geerligs et al., 2014; Raichlen et al., 2016; Long et al., 2017), and OCC (visual) (Yan et al., 2011; Damoiseaux et al., 2016; Chen et al., 2017; Zhang et al., 2017), appeared to increase in the early years and decrease in the later years, showing an inverted U shape over lifespan time. The functional connectivity between networks, such as the DMN-FPN (Geerligs et al., 2014) and FPN-OCC (Chen et al., 2017; Zhang et al., 2017), generally presented a U-shaped, age-related trend, decreasing in the early years, and increasing in the late years. The functional connectivity in each state changed with increasing age, following the same trends observed with the development of inherent networks or cognitive resources. This could also support the notion that micro-states involve intrinsic stable patterns of connectivity, which represent some specific cognitive resources.

### Age-Related Transitions Between Micro-States

The transitions between micro-states emerged as the fluctuations of functional connectivity. Although there was no difference in dwelling time between states, the transition time between micro-states significantly changed between groups, which could suggest that the transitions in micro-states are highly related to the development and aging process. The transition between states showed various patterns among the three groups. Some transition between states decreased from the young to the adult but increased from the adult to the old; other trainsitions increased in both two development procedure. These features suggested that the transition patterns might be intrinsic and that the transition strength could vary based on cognitive demand or deficit since age increasing.

When comparing the transition matrixes and the distance matrixes, the states with higher transition frequency showed shorter distances. This might suggest that the transition between micro-states or intrinsic connectivity patterns preferentially occur between the states with short distances. Based on the alterations in transition strength, we could speculate that the changes from one state to another state might be an avenue for information communication. There are both declines and growth in functional networks with age increasing, and these changes basically correspond to decreased function within intrinsic networks and increased function between intrinsic networks, respectively (Hagmann et al., 2010; La Corte et al., 2016). The various changes within or between networks provide the basis for micro-states transition, which can balance the cognitive decline and growth.

### The Intrinsic Variation in Each Micro-State

We suggested that the states were the basic functional connectivity patterns that could form certain combinations to respond to many kinds of situations, such as tasks and diseases. The transitions between the states or patterns are typical representations of the dynamic network changes that occur to adapt to many brain conditions. Age related changes in functional networks were actually a process of network decline, reorganization and transitions between states, according to results here and previous literatures.

To reveal the underlying features of the micro-states and to interpret what happened in the micro-sates with age increasing, we investigated each state with respect to the age-related variation for each state. The age-related variability analysis indicated that each micro-state demonstrated a different distribution of variability in the intrinsic networks. For example, S3 (state 3), which was close to that of the stationary connectivity, showed the highest variability in FPN, suggesting clear changes across the three groups. In addition, the distributions of the age-related variability in all states appeared to demonstrate a complementary pattern. The DMN showed higher variability in S1 and S2. The FPN presented high variation in S1 and S3. The fact that the OCC showed lower variation in all states, might be due to the higher aging related variation of FPN in other states (Davis et al., 2008). We suggested that the dynamic transitions between states could compensate for the age related variation in some intrinsic cognitive networks to balance the age-related changes in the different cognitive systems.

In fact, many previous studies (Spreng et al., 2010; Barber et al., 2013; Toussaint et al., 2014; Kennedy et al., 2015; Li et al., 2015; Grady et al., 2016) reported the aging related differences in the functional interactions among cognitive networks, such as the frontoparietal network and default mode network, and that constituted a mechanism of reorganization that served to balance the various age-related changes, including both decline and growth. When the micro-states involved stable connectivity patterns, similar to the intrinsic cognitive resources or functional networks, the early increases and late decreases observed in the current research were consistent with the literatures (Friston et al., 1996; Tomasi and Volkow, 2012; Betzel et al., 2014; Spreng et al., 2016; Keunen et al., 2017). Some reorganization and compensation of the functional networks were processed via the dynamic transitions between the stable connectivity patterns or micro-states. In fact, studies have found the dynamic reorganization (Sala-Llonch et al., 2012) and dynamic participation (Schaefer et al., 2014) of functional connectivity hubs in healthy brain networks also using the sliding window correlation method.

### Limitations and Perspective

There were several limitations that need further investigation in future work. First, the optimal number of clustering was a result of the data-driven method based on the current data analysis only. We thought there could be more specific micro-states when more detailed parcellations of the network and higher time resolution of the dynamic analysis were performed. This was similar to the modularity analysis in that the number of intrinsic networks could be defined in various scales. Second, there was a need to investigate the underlying processes of the transitions between micro-states in future research. In this paper, we revealed that the transitions between states were most likely an important mechanism of functional reorganization with age increasing. The inherent reasons for the transitions, as well as for the age-related changes, were still not clear.

We performed a dynamic functional connectivity analysis and provided a method for extracting the functional micro-states using dynamic analysis. The dynamic transitions between these micro-states could be highly related to the macroscopic dynamic changes in functional networks with age increasing. These states presented the basic and important functional connectivity patterns that could be combined with the dynamic changes in the whole functional networks. These types of connectivity patterns were similar to the network basis, such as motif, to spatially decompose brain network (Hutchison et al., 2013a; Sporns, 2013; Betzel et al., 2016). We think that this network analysis approach is well enough within the concepts of the emerging field of network medicine and network physiology (Ivanov et al., 2016). Constructing networks from medical images (in this case fMRI) is one kind of approach which is complementary to the signal analysis (Bartsch et al., 2015; Liu et al., 2015). This approach also highlights the interplay between network topology and function (Bashan et al., 2012).

## Conclusion

We have performed, to our knowledge, the first whole-brain characterization of age-related micro-states in the view of dynamic functional connectivity. The key finding was that the age-related changes in functional connectivity micro-states mainly occurred in the transitions between state. This could be used as distinctive characteristic markers or technique to observe the underlying neural activities in our brain systems and to reveal specific aging mechanism.

## Author Contributions

YC wrote this paper with the assistance of YL. YC, YL, XZ, and QW finished the date processing, feature analysis, and figures plotting. YC and PZ processed the statistical analysis. YC, XZ, and DM provided the original idea. XZ and DM sponsored this study.

## Conflict of Interest Statement

The authors declare that the research was conducted in the absence of any commercial or financial relationships that could be construed as a potential conflict of interest.

## Footnotes

1 http://fcon_1000.projects.nitrc.org/indi/pro/nki.html

2 http://rfmri.org/dpabi

3 https://www.fil.ion.ucl.ac.uk/spm/

## References

[B1] Allen E. A. Damaraju E. Plis S. M. Erhardt E. B. Eichele T. Calhoun V. D. (2014). Tracking whole-brain connectivity dynamics in the resting state. *Cereb. Cortex* 24 663–676. 10.1093/cercor/bhs352 23146964 PMC3920766

[B2] Barber A. D. Caffo B. S. Pekar J. J. Mostofsky S. H. (2013). Developmental changes in within- and between-network connectivity between late childhood and adulthood. *Neuropsychologia* 51 156–167. 10.1016/j.neuropsychologia.2012.11.011 23174403 PMC3543510

[B3] Bartsch R. P. Liu K. K. L. Bashan A. Ivanov P. C. (2015). Network physiology: How organ systems dynamically interact. *PLoS One* 10:e0142143. 10.1371/journal.pone.0142143 26555073 PMC4640580

[B4] Bashan A. Bartsch R. P. Kantelhardt J. W. Havlin S. Ivanov P. C. (2012). Network physiology reveals relations between network topology and physiological function. *Nat. Commun.* 3:1705. 10.1038/ncomms1705 22426223 PMC3518900

[B5] Betzel R. F. Avena-Koenigsberger A. Goñi J. He Y. de Reus M. A. Griffa A. (2016). Generative models of the human connectome. *Neuroimage* 124 1054–1064. 10.1016/j.neuroimage.2015.09.041 26427642 PMC4655950

[B6] Betzel R. F. Byrge L. He Y. Goñi J. Zuo X. N. Sporns O. (2014). Changes in structural and functional connectivity among resting-state networks across the human lifespan. *Neuroimage* 102 345–357. 10.1016/j.neuroimage.2014.07.067 25109530

[B7] Braun U. Schäfer A. Bassett D. S. Rausch F. Schweiger J. I. Bilek E. (2016). Dynamic brain network reconfiguration as a potential schizophrenia genetic risk mechanism modulated by NMDA receptor function. *Proc. Natl. Acad. Sci. U.S.A.* 113 12568–12573. 10.1073/pnas.1608819113 27791105 PMC5098640

[B8] Calhoun V. D. Miller R. Pearlson G. Adali T. (2014). The chronnectome: time-varying connectivity networks as the next frontier in fMRI data discovery. *Neuron* 84 262–274. 10.1016/j.neuron.2014.10.015 25374354 PMC4372723

[B9] Cao M. Wang J. H. Dai Z. J. Cao X. Y. Jiang L. L. Fan F. M. (2014). Topological organization of the human brain functional connectome across the lifespan. *Dev. Cogn. Neurosci.* 7 76–93. 10.1016/j.dcn.2013.11.004 24333927 PMC6987957

[B10] Chang C. Glover G. H. (2010). Time-frequency dynamics of resting-state brain connectivity measured with fMRI. *Neuroimage* 50 81–98. 10.1016/j.neuroimage.2009.12.011 20006716 PMC2827259

[B11] Chen Y. Wang W. Zhao X. Sha M. Liu Y. Zhang X. (2017). Age-related decline in the variation of dynamic functional connectivity: a resting state analysis. *Front. Aging Neurosci.* 9:203. 10.3389/fnagi.2017.00203 28713261 PMC5491557

[B12] Damoiseaux J. S. Viviano R. P. Yuan P. Raz N. (2016). Differential effect of age on posterior and anterior hippocampal functional connectivity. *Neuroimage* 133 468–476. 10.1016/j.neuroimage.2016.03.047 27034025 PMC4889536

[B13] Davis S. W. Dennis N. A. Daselaar S. M. Fleck M. S. Cabeza R. (2008). Qué PASA? The posterior-anterior shift in aging. *Cereb. Cortex* 18 1201–1209. 10.1093/cercor/bhm155 17925295 PMC2760260

[B14] Di X. Biswal B. B. (2013). Dynamic brain functional connectivity modulated by resting-state networks. *Brain Struct. Funct.* 220 37–46. 10.1007/s00429-013-0634-3 25713839 PMC3980132

[B15] Dosenbach N. U. F. Nardos B. Cohen A. L. Fair D. A Power D. Church J. A. (2011). Prediction of individua brain maturity using fMRI. *Science (80-)* 329 1358–1361. 10.1126/science.1194144.Prediction PMC3135376 20829489

[B16] Douaud G. Groves A. R. Tamnes C. K. Westlye L. T. Duff E. P. Engvig A. (2014). A common brain network links development, aging, and vulnerability to disease. *Proc. Natl. Acad. Sci. U.S.A.* 111 17648–17653. 10.1073/pnas.1410378111 25422429 PMC4267352

[B17] Du Y. Pearlson G. D. Yu Q. He H. Lin D. Sui J. (2016). Interaction among subsystems within default mode network diminished in schizophrenia patients: a dynamic connectivity approach. *Schizophr. Res.* 170 55–65. 10.1016/j.schres.2015.11.021 26654933 PMC4707124

[B18] Feng G. Chen H. C. Zhu Z. He Y. Wang S. (2015). Dynamic brain architectures in local brain activity and functional network efficiency associate with efficient reading in bilinguals. *Neuroimage* 119 103–118. 10.1016/j.neuroimage.2015.05.100 26095088

[B19] Friston K. J. Williams S. Howard R. Frackowiak R. S. J. Turner R. (1996). Movement-related effects in fMRI time-series. *Magn. Reson. Med.* 35 346–355. 10.1002/mrm.1910350312 8699946

[B20] Geerligs L. Renken R. J. Saliasi E. Maurits N. M. Lorist M. M. (2014). A brain-wide study of age-related changes in functional connectivity. *Cereb. Cortex* 2 1–13. 10.1093/cercor/bhu012 24532319

[B21] Gonzalez-Castillo J. Hoy C. W. Handwerker D. A. Robinson M. E. Buchanan L. C. Saad Z. S. (2015). Tracking ongoing cognition in individuals using brief, whole-brain functional connectivity patterns. *Proc. Natl. Acad. Sci. U.S.A.* 112 8762–8767. 10.1073/pnas.1501242112 26124112 PMC4507216

[B22] Grady C. Sarraf S. Saverino C. Campbell K. (2016). Age differences in the functional interactions among the default, frontoparietal control, and dorsal attention networks. *Neurobiol. Aging* 41 159–172. 10.1016/j.neurobiolaging.2016.02.020 27103529

[B23] Hagmann P. Cammoun L. Gigandet X. Gerhard S. Ellen Grant P. Wedeen V. (2010). MR connectomics: principles and challenges. *J. Neurosci. Methods* 194 34–45. 10.1016/j.jneumeth.2010.01.014 20096730

[B24] Hedden T. Gabrieli J. D. E. (2004). Insights into the ageing mind: a view from cognitive neuroscience. *Nat. Rev. Neurosci.* 5 87–96. 10.1038/nrn1323 14735112

[B25] Hutchison R. M. Womelsdorf T. Allen E. A. Bandettini P. A. Calhoun V. D. Corbetta M. (2013a). Dynamic functional connectivity: promise, issues, and interpretations. *Neuroimage* 80 360–378. 10.1016/j.neuroimage.2013.05.079 23707587 PMC3807588

[B26] Hutchison R. M. Womelsdorf T. Gati J. S. Everling S. Menon R. S. (2013b). Resting-state networks show dynamic functional connectivity in awake humans and anesthetized macaques. *Hum. Brain Mapp.* 34 2154–2177. 10.1002/hbm.22058 22438275 PMC6870538

[B27] Ivanov P. C. H. Liu K. K. L. Bartsch R. P. (2016). Focus on the emerging new fields of network physiology and network medicine. *New J. Phys.* 18:100201. 10.1088/1367-2630/18/10/100201 30881198 PMC6415921

[B28] Kaiser R. H. Whitfield-Gabrieli S. Dillon D. G. Goer F. Beltzer M. Minkel J. (2015). Dynamic resting-state functional connectivity in major depression. *Neuropsychopharmacology* 41 1822–1830. 10.1038/npp.2015.352 26632990 PMC4869051

[B29] Kennedy K. M. Rodrigue K. M. Bischof G. N. Hebrank A. C. Reuter-Lorenz P. A. Park D. C. (2015). Age trajectories of functional activation under conditions of low and high processing demands: an adult lifespan fMRI study of the aging brain. *Neuroimage* 104 21–34. 10.1016/j.neuroimage.2014.09.056 25284304 PMC4252495

[B30] Keunen K. Counsell S. J. Benders M. J. (2017). The emergence of functional architecture during early brain development. *Neuroimage* 160 2–14. 10.1016/j.neuroimage.2017.01.047 28111188

[B31] La Corte V. Sperduti M. Malherbe C. Vialatte F. Lion S. Gallarda T. (2016). Cognitive decline and reorganization of functional connectivity in healthy aging: the pivotal role of the salience network in the prediction of age and cognitive performances. *Front. Aging Neurosci.* 8:204. 10.3389/fnagi.2016.00204 27616991 PMC5003020

[B32] Lehmann D. (1990). “Brain electric microstates and cognition: the atoms of thought,” in *Machinery of the Mind*, eds John E. R. Harmony T. Prichep L. S. Valdés-Sosa M. Valdés-Sosa P. A. (Boston, MA: Birkhäuser). 10.1007/978-1-4757-1083-0_10

[B33] Leonardi N. Van De Ville D. (2015). On spurious and real fluctuations of dynamic functional connectivity during rest. *Neuroimage* 104 430–436. 10.1016/j.neuroimage.2014.09.007 25234118

[B34] Li Y. Li C. Wu Q. Xu Z. Kurata T. Ohno S. (2015). Decreased resting-state connections within the visuospatial attention-related network in advanced aging. *Neurosci. Lett.* 597 13–18. 10.1016/j.neulet.2015.03.047 25817360

[B35] Liu K. K. L. Bartsch R. P. Lin A. Mantegna R. N. Ivanov P. C. (2015). Plasticity of brain wave network interactions and evolution across physiologic states. *Front. Neural Circuits* 9:62. 10.3389/fncir.2015.00062 26578891 PMC4620446

[B36] Long X. Benischek A. Dewey D. Lebel C. (2017). Age-related functional brain changes in young children. *Neuroimage* 155 322–330. 10.1016/j.neuroimage.2017.04.059 28461057

[B37] Mayer A. R. Ling J. M. Allen E. A. Klimaj S. D. Yeo R. A. Hanlon F. M. (2015). Static and dynamic intrinsic connectivity following mild traumatic brain injury. *J. Neurotrauma* 32 1046–1055. 10.1089/neu.2014.3542 25318005 PMC4504345

[B38] Park D. C. McDonough I. M. (2013). The dynamic aging mind: revelations from functional neuroimaging research. *Perspect. Psychol. Sci.* 8 62–67. 10.1177/1745691612469034 26172252

[B39] Pascual-Marqui R. D. (1995). Segmentation of brain electrical activity into microstates: model estimation and validation. *IEEE Trans.* 42 1–24. 10.1109/10.391164 7622149

[B40] Power J. D. Barnes K. A. Snyder A. Z. Schlaggar B. L. Petersen S. E. (2011). Spurious but systematic correlations in functional connectivity MRI networks arise from subject motion. *NeuroImage* 59 2142–2154. 10.1016/j.neuroimage.2011.10.018 22019881 PMC3254728

[B41] Qin J. Chen S.-G. Hu D. Zeng L.-L. Fan Y.-M. Chen X.-P. (2015). Predicting individual brain maturity using dynamic functional connectivity. *Front. Hum. Neurosci.* 9:418. 10.3389/fnhum.2015.00418 26236224 PMC4503925

[B42] Raichlen D. A. Bharadwaj P. K. Fitzhugh M. C. Haws K. A. Torre G.-A. Trouard T. P. (2016). Differences in resting state functional connectivity between young adult endurance athletes and healthy controls. *Front. Hum. Neurosci.* 10:610. 10.3389/fnhum.2016.00610 28018192 PMC5147411

[B43] Rashid B. Damaraju E. Pearlson G. D. Calhoun V. D. (2014). Dynamic connectivity states estimated from resting fMRI Identify differences among Schizophrenia, bipolar disorder, and healthy control subjects. *Front. Hum. Neurosci.* 8:897. 10.3389/fnhum.2014.00897 25426048 PMC4224100

[B44] Reeve A. Simcox E. Turnbull D. (2014). Ageing and Parkinson’s disease: why is advancing age the biggest risk factor? *Ageing Res. Rev.* 14 19–30. 10.1016/j.arr.2014.01.004 24503004 PMC3989046

[B45] Reuter-Lorenz P. A. Campbell K. A. (2008). Neurocognitive ageing and the Compensation Hypothesis. *Curr. Dir. Psychol. Sci.* 17 177–182. 10.1111/j.1467-8721.2008.00570.x

[B46] Sakoglu Ü. Pearlson G. D. Kiehl K. A. Wang Y. M. Michael A. M. Calhoun V. D. (2010). A method for evaluating dynamic functional network connectivity and task-modulation: application to schizophrenia. *Magn. Reson. Mater. Phys. Biol. Med.* 23 351–366. 10.1007/s10334-010-0197-8 20162320 PMC2891285

[B47] Sala-Llonch R. Arenaza-Urquijo E. M. Valls-Pedret C. Vidal-Piñeiro D. Bargalló N. Junqué C. (2012). Dynamic functional reorganizations and relationship with working memory performance in healthy aging. *Front. Hum. Neurosci.* 6:152. 10.3389/fnhum.2012.00152 22701409 PMC3369258

[B48] Schaefer A. Margulies D. S. Lohmann G. Gorgolewski K. J. Smallwood J. Kiebel S. J. (2014). Dynamic network participation of functional connectivity hubs assessed by resting-state fMRI. *Front. Hum. Neurosci.* 8:195. 10.3389/fnhum.2014.00195 24860458 PMC4018560

[B49] Siman-Tov T. Bosak N. Sprecher E. Paz R. Eran A. Aharon-Peretz J. (2017). Early age-related functional connectivity decline in high-order cognitive networks. *Front. Aging Neurosci.* 8:330. 10.3389/fnagi.2016.00330 28119599 PMC5223363

[B50] Sporns O. (2013). The human connectome: origins and challenges. *Neuroimage* 80 53–61. 10.1016/j.neuroimage.2013.03.023 23528922

[B51] Spreng R. N. Stevens W. D. Chamberlain J. P. Gilmore A. W. Schacter D. L. (2010). Default network activity, coupled with the frontoparietal control network, supports goal-directed cognition. *Neuroimage* 53 303–317. 10.1016/j.neuroimage.2010.06.016 20600998 PMC2914129

[B52] Spreng R. N. Stevens W. D. Viviano J. D. Schacter D. L. (2016). Attenuated anticorrelation between the default and dorsal attention networks with aging: evidence from task and rest. *Neurobiol. Aging* 45 149–160. 10.1016/j.neurobiolaging.2016.05.020 27459935 PMC5003045

[B53] Tomasi D. Volkow N. D. (2012). Aging and functional brain networks. *Mol. Psychiatry* 17 471–558. 10.1038/mp.2011.81.Aging 21727896 PMC3193908

[B54] Toussaint P. J. Maiz S. Coynel D. Doyon J. Messe A. de Souza L. C. (2014). Characteristics of the default mode functiéonal connectivity in normal ageing and Alzheimer’s disease using resting state fMRI with a combined approach of entropy-based and graph theoretical measurements. *Neuroimage* 101 778–786. 10.1016/j.neuroimage.2014.08.003 25111470

[B55] Vinette S. A. Bray S. (2015). Variation in functional connectivity along anterior-to-posterior intraparietal sulcus, and relationship with age across late childhood and adolescence. *Dev. Cogn. Neurosci.* 13 32–42. 10.1016/j.dcn.2015.04.004 25951196 PMC6989812

[B56] Wee C. Y. Yang S. Yap P. T. Shen D. (2016). Sparse temporally dynamic resting-state functional connectivity networks for early MCI identification. *Brain Imaging Behav.* 10 342–356. 10.1007/s11682-015-9408-2 26123390 PMC4692725

[B57] Whalley L. J. Deary I. J. Appleton C. L. Starr J. M. (2004). Cognitive reserve and the neurobiology of cognitive aging. *Ageing Res. Rev.* 3 369–382. 10.1016/j.arr.2004.05.001 15541707

[B58] Xu L. Wu X. Chen K. Yao L. (2015). Multi-modality sparse representation-based classification for Alzheimer’s disease and mild cognitive impairment. *Comput. Methods Programs Biomed.* 122 182–190. 10.1016/j.cmpb.2015.08.004 26298855

[B59] Yan C. G. Cheung B. Kelly C. Colcombe S. Craddock R. C. Di Martino A. (2013). A comprehensive assessment of regional variation in the impact of head micromovements on functional connectomics. *Neuroimage* 76 183–201. 10.1016/j.neuroimage.2013.03.004 23499792 PMC3896129

[B60] Yan C. G. Wang X. D. Zuo X. N. Zang Y. F. (2016). DPABI: data processing & analysis for (resting-state) brain imaging. *Neuroinformatics* 14 339–351. 10.1007/s12021-016-9299-4 27075850

[B61] Yan L. Zhuo Y. Wang B. Wang D. J. J. (2011). Loss of coherence of low frequency fluctuations of BOLD FMRI in visual cortex of healthy aged subjects. *Open Neuroimag. J.* 5 105–111. 10.2174/1874440001105010105 22216081 PMC3245404

[B62] Zalesky A. Fornito A. Cocchi L. Gollo L. L. Breakspear M. (2014). Time-resolved resting-state brain networks. *Proc. Natl. Acad. Sci. U.S.A.* 111 10341–10346. 10.1073/pnas.1400181111 24982140 PMC4104861

[B63] Zhang H. Lee A. Qiu A. (2017). A posterior-to-anterior shift of brain functional dynamics in aging. *Brain Struct. Funct.* 222 3665–3676. 10.1007/s00429-017-1425-z 28417233

